# Real-world analysis of the efficacy and safety of nusinersen in pediatric patients with spinal muscular atrophy

**DOI:** 10.1186/s13023-025-03603-9

**Published:** 2025-02-26

**Authors:** Wenjing Li, Qin Zhang, Hongjun Miao, Jin Xu

**Affiliations:** 1 Department of Pharmacy, Children’s Hospital of Nanjing Medical University, 72 Guangzhou Road, Nanjing, 210008 China; 2 Emergency Department/Intensive Care Unit, Children’s Hospital of Nanjing Medical University, Nanjing, 210008 China

**Keywords:** Spinal muscular atrophy (SMA), Nusinersen, Motor function, Adverse reactions, Pharmacoeconomics

## Abstract

**Background:**

Spinal muscular atrophy (SMA) is a rare neurodegenerative disease that significantly affects multiple systems in children. Nusinersen, the first approved treatment for SMA, enhances SMN protein production by targeting the RNA splicing site of the SMN2 gene, thus improving motor function. However, the high cost of nusinersen treatment raises concerns about its economic feasibility.

**Methods:**

This study retrospectively analyzed clinical data of 42 pediatric SMA patients treated with nusinersen from January 2022 to October 2024 at our hospital. We assessed the efficacy, safety, and economic impact of nusinersen in different SMA types. Motor function was evaluated using the CHOP-INTEND, HINE-2, HFMSE, and RULM scales. Safety was assessed based on adverse reactions and events, and economic evaluation considered total treatment costs and average cost per injection.

**Results:**

Nusinersen significantly improved motor function in SMA patients, especially in type I patients, who showed notable increases in CHOP-INTEND and HINE-2 scores. The RULM score had the highest increase among type II patients, while improvements were relatively lower in type III patients. Regarding safety, the incidence of adverse events was 40.48%, with fever being the most common adverse reaction, occurring in 36.36% of cases. Economic analysis indicated that the total treatment cost was highest for type III patients, though the cost differences among types were not statistically significant (*P* > 0.05).

**Conclusion:**

Nusinersen demonstrated significant clinical efficacy and favorable safety in pediatric SMA patients, with improved economic feasibility after insurance coverage. Our findings support early SMA screening and presymptomatic nusinersen administration to maximize therapeutic benefits. Further multicenter, large-sample, long-term follow-up studies are warranted to validate and expand upon these findings.

Spinal muscular atrophy (SMA) is a rare, autosomal recessive genetic neuromuscular disorder [1]. It is typically caused by the homozygous deletion or other pathogenic variants of the survival motor neuron 1 (SMN1) gene located on chromosome 5q13.2, leading to insufficient levels of SMN protein. This deficiency results in the degeneration of anterior horn α-motor neurons in the spinal cord and lower brainstem motor nuclei. Clinically, SMA manifests as progressive, symmetrical muscle weakness and atrophy, primarily affecting the proximal muscles of the limbs [2]. As the disease progresses, patients may gradually lose the ability to walk, sit independently, and swallow, eventually leading to respiratory muscle involvement. This can culminate in progressive respiratory failure due to respiratory muscle weakness, which may ultimately result in death [3].

The incidence of SMA is approximately 1 in 11,000, with a carrier rate ranging from 1 in 72 to 1 in 47 [4]. The incidence in newborns is about 0.01%, making SMA a major genetic cause of infant mortality [5]. In May 2018, SMA was included in the first list of rare diseases in China [6, 7] and is the most prevalent rare disease in the country [8]. SMA can be classified into four types based on the age of onset and the maximum motor function achieved by the patient [9]. Types I to III are pediatric forms, while Type IV is classified as adult onset. Type I accounts for approximately 45% of all SMA cases [10]. Symptoms typically appear within the first six months of birth and include severe hypotonia and lack of head control. The disease progresses rapidly, with early involvement of respiratory muscles leading to respiratory weakness. Without treatment, most patients do not survive beyond the age of two due to respiratory failure [11, 12]. Type II represents about 30–40% of cases [10], with onset occurring between six to eighteen months of age. Patients can sit but are unable to walk, and about 70% survive to age 25. Type III accounts for approximately 20% of cases [10], with onset after eighteen months in childhood. Patients can walk, but they may eventually lose this ability as the disease progresses; however, it typically does not significantly affect life expectancy. Type IV has an adult onset, with normal motor milestones and relatively slow progression, often presenting only with proximal weakness in the lower limbs, which does not impact life expectancy. Additionally, type 0 refers to patients who present with symptoms in utero or at birth, lacking any motor milestones, and often resulting in stillbirth or death shortly after birth.

As the disease progresses, the muscle weakness associated with spinal muscular atrophy (SMA) can affect various systems. Therefore, the treatment of SMA often requires multidisciplinary assessment and comprehensive management. Pharmacological treatment aims to increase the expression levels of SMN protein. In December 2016, nusinersen received FDA approval for the treatment of children and adults with SMA, becoming the first drug globally approved for this condition [13]. Nusinersen is an antisense oligonucleotide that targets the critical splicing silencer N1 sequence within the splice site of intron 7 of the SMN2 gene, leading to upregulation of SMN protein expression and therapeutic effects [14]. To date, over 11,000 SMA patients have been treated with nusinersen, further confirming its safety and efficacy [15]. In April 2019, nusinersen injection was officially launched in China as the first drug approved for the treatment of SMA. Due to its later introduction in the country and high cost, there have been few related clinical application reports. In December 2021, nusinersen injection was included in the national medical insurance catalog, becoming the first SMA gene therapy drug covered by national insurance, significantly improving drug accessibility. Since 2022, our hospital has gradually begun treating pediatric patients with nusinersen.

This study employs a retrospective analysis method to collect real-world data from pediatric patients diagnosed with SMA who received nusinersen treatment at our hospital between January 2022 and October 2024. The analysis aims to evaluate the efficacy and safety of nusinersen in treating children with SMA, providing valuable insights for the clinical management of pediatric SMA patients.

## Materials and methods

### Study population

This retrospective study analyzed the medical records of 42 pediatric patients diagnosed with SMA who received intrathecal nusinersen injections at our hospital from January 2022 to October 2024. The study was approved by the Ethics Committee of Children’s Hospital of Nanjing Medical University, and informed consent from patients was waived.

### Inclusion and exclusion criteria

Inclusion criteria: (1) Diagnosis consistent with the 2019 Expert Consensus on Multidisciplinary Management of SMA; (2) Age ≤ 18 years; (3) Nusinersen as the initial monotherapy treatment with a dosage of 12 mg (5 mL) per injection; (4) Availability of at least one outcome measure, including the Children’s Hospital of Philadelphia Infant Test of Neuromuscular Disorders (CHOP INTEND) [16], the Revised Upper Limb Module (RULM) score, the Hammersmith Functional Motor Scale Expanded (HFMSE) [17], the HINE2 score, and the Developmental Assessment of Young Children [18, 19].

Exclusion criteria: (1) Patients who do not meet the above inclusion criteria; (2) Patients with missing clinical data.

### Collection of clinical data

#### General information

Including the patient’s gender, age, and date of birth.

#### Clinical data

Patient medical records were reviewed to gather information on the age of onset, clinical manifestations (including normal or abnormal eating, language abilities, strength of upper and lower limbs, ability to sit independently, head control stability, ability to stand, ability to walk, ability to raise both upper limbs, voluntary grasping with hands, lower limb mobility, and ability to lift legs off the bed). Additional data collected included electromyography (EMG) results, genetic testing data and results (SMN1 genotype), SMA clinical classification, age at initiation of nusinersen treatment, timing of each intrathecal injection, length of hospital stay for each injection, hospitalization costs, and laboratory test results.

Laboratory tests included complete blood count, procalcitonin (PCT), coagulation parameters, liver and kidney function, creatine kinase-MB (CK-MB), cerebrospinal fluid (CSF) biochemistry, and 25-hydroxyvitamin D levels.

Specifically, complete blood count parameters included C-reactive protein (CRP), white blood cell count (WBC), neutrophils percentage (N%), lymphocytes percentage (L%), platelet count (PLT), and red blood cell count (RBC). Coagulation parameters included international normalized ratio (INR), thrombin time, activated partial thromboplastin time (aPTT), and D-dimer. Liver function parameters included alanine aminotransferase (ALT), aspartate aminotransferase (AST), and gamma-glutamyl transferase (GGT). Kidney function parameters included blood urea nitrogen (BUN), uric acid (UA), and creatinine (Cr). CSF biochemistry included glucose, chloride, protein, and lactate dehydrogenase levels.

#### Outcome measures

Treatment effectiveness was evaluated using CHOP INTEND, RULM, HFMSE, and HINE2 scores, as well as an assessment of maximum motor milestones, including milestone movements such as voluntary grasping, leg lifting, head control, rolling, sitting independent sitting, crawling, standing, and walking. Treatment effectiveness was assessed at each intrathecal injection session, with clinical effectiveness defined as improvement in at least one outcome measure.

The CHOP INTEND score reflects the severity of the disease and is applicable to infants and patients unable to sit independently [20]. An increase of ≥ 4 points in the CHOP INTEND score is considered a clinically meaningful improvement.

RULM is primarily used to assess upper limb function, evaluating shoulder, elbow, wrist, and hand movements in the dominant arm across 19 activities related to daily life, with each item scored from 0 to 2 points. The total score for a single upper limb is 37 points, with the dominant side score used for analysis. Higher scores indicate better functionality, and an increase of ≥ 2 points in the RULM score is considered clinically meaningful.

HINE-2 is primarily used to assess developmental milestones in children aged 2 months to 2 years, evaluating functions such as head control, sitting, voluntary grasping, kicking in the supine position, rolling, crawling, standing, and walking. An increase of ≥ 2 points in the HINE-2 score is considered clinically meaningful.

#### Adverse reactions and adverse events

All medical events causing discomfort in patients after administration were recorded. Referring to the nusinersen sodium package insert, any adverse reactions not mentioned in the insert were considered new adverse drug reactions.

### Statistical methods

This study utilized SPSS version 26.0 for statistical analysis. Normally distributed continuous data are presented as mean ± standard deviation (x̄ ± s). For continuous data not conforming to a normal distribution or for ordinal data, the median (25th percentile, 75th percentile) and frequency (n, %) are reported. Group comparisons were conducted using the t-test for continuous data and the chi-squared (χ²) test for categorical data. A p-value of < 0.05 was considered statistically significant.

## Results

### General data

A total of 42 children diagnosed with SMA and treated with intrathecal injections of nusinersen sodium were included in this study. Among them, 19 (45.2%) were male and 23 (54.8%) were female, with no statistically significant difference in gender distribution. The median age at disease onset was 8.0 months (6.0, 15.0), the median age at genetic confirmation was 18.0 months (8.0, 23.0), and the median number of nusinersen treatments was 10.0 (7.3, 11.8).

Based on clinical classifications, there were 12 children (28.6%) with type I SMA, with 7 males (58.3%) and 5 females (41.7%), showing no statistically significant gender difference. The average age of onset for type I patients was 4.2 ± 1.5 months, with genetic diagnosis at 7.3 ± 5.7 months, and initiation of nusinersen treatment at 34.3 ± 21.7 months. For type II SMA, there were 26 cases (61.9%), with 10 males (38.5%) and 16 females (61.5%), also with no significant gender difference. The average age of onset for type II patients was 11.4 ± 4.0 months, genetic diagnosis at 84.8 ± 290.5 months, and treatment initiation at 127.3 ± 283.1 months. There were 4 cases (9.5%) of type III SMA, with 2 males (50.0%) and 2 females (50.0%), with no significant gender difference. The average age of onset for type III patients was 47.0 ± 58.7 months, genetic diagnosis at 56.5 ± 66.4 months, and treatment initiation at 85.5 ± 53.9 months.

Among these 42 children, 9 cases (21.4%) initially presented with muscle weakness, 13 (31.0%) with developmental delays, 6 (14.3%) with unstable head control, 4 (9.5%) with inability to roll over, 7 (16.7%) with inability to sit independently, 9 (21.4%) with inability to stand, and 11 (26.2%) with inability to walk. Electrodiagnostic studies indicated neurogenic changes in 27 cases (64.3%) and myopathic changes in 1 case (2.4%), with no abnormal findings in the remaining 14 cases (33.3%). Genetic testing showed SMN1 exon 7 and 8 deletions in 32 cases (76.2%), SMN1 exon 7 deletion in 7 cases (16.7%), and missing genetic results in 3 cases (7.1%). General data for children with different SMA clinical subtypes are provided in Table 1.

**Table 1:** General information of SMA patients with different clinical types treated with nusinersen sodium [M (Q1, Q3)]

Variable	Type I (*n* = 12)	Type II (*n* = 26)	Type III (*n* = 4)	Total (*n* = 42)	*P*
Gender, *n*(%)					0.383
Male	7(58.3)	10(38.5)	2(50.0)	19(45.2)	0.509
Female	5(41.7)	16(61.5)	2(50.0)	23(54.8)	0.509
Age at onset (months)	4.0(3.8,5.3)	12.0(8.0,15.0)	18.5(17.5,48.0)	8.0(6.0,15.0)	-
Symptoms at onset, *n*(%)					
Muscle weakness	2(16.7)	6(23.1)	1(25.0)	9(21.4)	0.890
Motor development delay	4(33.3)	9(34.6)	0(0)	13(31.0)	0.370
Unstable head control	5(41.7)	1(3.8)	0(0)	6(14.3)	0.006^*^
Inability to roll over	2(16.7)	2(7.7)	0(0)	4(9.5)	0.540
Inability to sit independently	2(16.7)	5(19.2)	0(0)	7(16.7)	0.630
Inability to stand	0(0)	8(30.8)	1(25.0)	9(21.4)	0.098
Inability to walk	0(0)	9(34.6)	2(50.0)	11(26.2)	0.041^*^
EMG results, *n*(%)					
Neurogenic lesion	6(50.0)	17(65.4)	4(100.0)	27(64.3)	0.192
Myogenic lesion	0(0)	1(3.8)	0(0)	1(2.4)	0.730
No EMG results	6(50.0)	8(30.8)	0(0)	14(33.3)	0.167
Age at genetic confirmation (months)	6.0(4.5,8.0)	18(14.3,22.8)	27.0(25.5,91.5)	18.0(8.0,23.0)	-
SMN1gene test results, *n*(%)					
Exon 7 homozygous deletion	0(0)	7(26.9)	0(0)	7(16.7)	0.075
Exons 7 and 8 homozygous deletion	8(66.7)	16(61.5)	3(75.0)	27(64.3)	0.854
Exon 7 homozygous and exon 8 heterozygous deletion	0(0)	3(11.5)	0(0)	3(7.1)	0.370
Exons 7 and 8 heterozygous deletion	1(8.3)	0(0)	1(25.0)	2(4.8)	0.072
Genetic result missing	3(25.0)	0(0)	0(0)	3(7.1)	0.018^*^
Disease duration (months)	84.0(56.5,87.0)	73.0(36.0,114.0)	61.0(35.0,88.5)	84.0(39.0,106.5)	0.685
Age at initial nusinersen treatment (months)	33.5(18.8,52.0)	89.0(28.0,91.0)	76.5(60.0,102.0)	-	< 0.001^*^

Data are presented as medians with interquartile ranges [M (Q1, Q3)] or as numbers with percentages *n* (%).

*P*-values were calculated for statistical significance, with *P < 0.05 indicating significant differences.

### Efficacy of nusinersen sodium injection for the treatment of SMA

Nusinersen cannot cross the blood-brain barrier and needs to be administered intrathecally. Because nusinersen will degrade over time, it needs to be administered once every four months for lifelong treatment. In our study, all children were evaluated for treatment efficacy on the day of the first nusinersen sodium treatment (Day 0), as well as at 2, 6, 10, 14, 18, 22, 26, 30, and 34 months post-treatment. The treatment efficacy was evaluated based on the patients’ clinical responses, and the results indicated significant variations in efficacy across different types of SMA. In patients diagnosed with type I SMA, the treatment was deemed effective on the day of the initial injection, as well as at the 2-month and 18-month follow-up points; For type II SMA patients, the treatment was effective on the day of the first injection, at 2 months, and at 6 months post-treatment; In type III SMA patients, treatment was considered effective only on the day of treatment and at the 2-month follow-up. After the initial treatment, the treatment efficacy rate for type II SMA patients was higher than that for type I and type III SMA patients. Two months after treatment, type III SMA patients had a higher treatment efficacy rate than type I and type II SMA patients. However, these differences were not statistically significant (*P* > 0.05). The results are presented in Table 2.

**Table 2:** Number and proportion of effective cases following nusinersen treatment, *n* (%)

SMA type	Nusinersen treatment time (months)				Total (*n* = 42)
	0	2	6	18	
Type I (*n* = 12)	2(16.7)	5(41.7)	-	1(8.3)	8(66.7)
Type II (*n* = 26)	8(30.8)	6(23.1)	2(7.7)	-	16(61.5)
Type III (*n* = 4)	1(25.0)	2(50.0)	-	-	3(75.0)
Total(*n* = 42)	11(26.2)	13(31.0)	2(4.8)	1(2.4)	27(64.3)
*P*	0.654	0.354	0.524	0.278	0.854

#### CHOP INTEND score

A total of 23 patients (85.2%) showed clinical effectiveness based on the CHOP INTEND score after receiving nusinersen treatment. Among these, 7 type I SMA patients (58.3%), 15 type II SMA patients (57.7%), and 1 type III SMA patient (25.0%) demonstrated clinical improvement. Furthermore, among the 23 clinically effective patients, 17 exhibited clinically meaningful improvement in their scores. Type II SMA patients had the highest proportion of clinically meaningful improvement, accounting for 86.67%, followed by 4 type I SMA patients (57.14%). Notably, none of the type III SMA patients achieved a clinically meaningful improvement in their CHOP INTEND score. These results are presented in Table 3.

**Table 3:** Clinical efficacy and clinically meaningful improvement in SMA patients after nusinersen treatment, *n* (%)

Type	CHOP INTEND score		RULM score		HFMSE score		HINE2 score		Motor milestones response
	Clinical effective	Clinically meaningful improvement	Clinical effective	Clinically meaningful improvement	Clinical effective	Clinically meaningful improvement	Clinical effective	Clinically meaningful improvement	
I	7(58.3)	4(57.14)	3(25.0)	2(66.67)	2(16.7)	2(100.0)	4(33.3)	3(75.0)	4(33.3)
II	15(57.7)	13(86.67)	10(38.5)	10(100.0)	13(50.0)	11(84.62)	13(50.0)	12(92.31)	13(50.0)
III	1(25.0)	0(0)	1(25.0)	1(100.0)	3(75.0)	2(66.67)	1(25.0)	0(0)	3(75.0)
Total	23(85.2)	17(73.91)	14(51.9)	13(92.86)	18(66.7)	15(83.33)	18(66.7)	15(83.33)	20(74.1)
*P*	0.453	0.077	0.668	0.139	0.061	0.602	0.471	0.051	0.326

In terms of score change, both type I and type II SMA patients showed an average increase of 8 points in their CHOP INTEND scores after treatment. In contrast, type III SMA patients had a relatively modest increase, with an average of 1 point. The detailed score changes can be seen in Table 4.

**Table 4:** Improvement in various scores after nusinersen treatment in SMA patients [M (Q1, Q3)]

Type	CHOP INTEND score	RULM score	HFMSE score	HINE2 score
I (*n* = 12)	8(2.0,13.5)	2(1.5,3.0)	6.5(4.8,8.2)	3.5(2.5,5.0)
II (*n* = 26)	8(4.0,10.5)	5.5(3.3,8.8)	4(3.0,10.0)	3(2.0,4.0)
III (*n* = 4)	1(1.0,1.0)	2(2.0,2.0)	4(3.0,7.0)	1(1.0,1.0)
Total (*n* = 42)	8(3.5,10.5)	4(2.3,7.5)	4(3.0,10.0)	3(2.0,4.0)
*P*	0.304	0.086	0.914	0.308

#### RULM score

A total of 14 patients (51.9%) showed clinical effectiveness based on the RULM score following nusinersen treatment. Among these, 3 type I SMA patients (25.0%), 10 type II SMA patients (38.5%), and 1 type III SMA patient (25.0%) demonstrated clinical improvement. Of the 14 clinically effective patients, 13 experienced clinically meaningful improvement. Specifically, all clinically effective type II and III SMA patients showed meaningful improvement, while 2 type I SMA patients (66.67%) achieved clinically significant progress. The results are detailed in Table 3.

Type II SMA patients experienced the most significant increase in their RULM scores, with an average increase of 5.5 points. Both type I and type III SMA patients showed an average increase of 2 points in their RULM scores after treatment. These findings are summarized in Table 4.

#### HFMSE score

A total of 18 patients (66.7%) demonstrated clinical effectiveness based on the HFMSE score after receiving nusinersen treatment. Among these, 2 type I SMA patients (16.7%), 13 type II SMA patients (50.0%), and 3 type III SMA patients (75.0%) showed clinical improvement. Of the 18 clinically effective patients, 15 exhibited clinically meaningful improvement. Type II SMA patients had the highest proportion of meaningful improvement, with 84.62%, followed by 2 type I and 2 type III SMA patients. The results are presented in Table 3.

Following treatment, type I SMA patients showed the largest increase in their HFMSE scores, with an average increase of 6.5 points. Both type II and type III SMA patients had an average increase of 4 points in their HFMSE scores. These results are displayed in Table 4.

#### HINE2 score

A total of 18 SMA patients (66.7%) demonstrated clinical efficacy based on the HINE2 score after treatment with nusinersen. Among these, 4 type I SMA patients (33.3%), 13 type II SMA patients (50.0%), and 1 type III SMA patient (25.0%) showed clinical improvement. Of the 18 clinically effective patients, 15 showed clinically meaningful improvement. Type II SMA patients had the highest proportion of meaningful improvement (92.31%), followed by 3 type I SMA patients (75.0%). No type III SMA patients showed a clinically meaningful improvement in their HINE2 scores. The results are provided in Table 3.

In terms of score improvement, type I SMA patients showed the highest average increase of 3.5 points in their HINE2 scores. Type II SMA patients had an average increase of 3 points, while type III SMA patients had an average increase of 1 point. The data can be found in Table 4.

#### Motor milestones response

A total of 20 patients (74.1%) demonstrated clinical effectiveness in terms of motor milestones response after receiving nusinersen treatment. Among these, 4 type I SMA patients (33.3%), 13 type II SMA patients (50.0%), and 3 type III SMA patients (75.0%) showed clinical improvement. The results are summarized in Table 3.

### Safety of nusinersen sodium injection for the treatment of SMA

Nusinersen has shown significant improvements in the motor function of patients with various types of SMA, with good safety and tolerance profiles. Among the 42 SMA children treated, 17 patients (40.48%) experienced adverse drug reactions, leading to a total of 33 adverse events. These events included fever, excessive salivation, cough, vomiting, elevated white blood cell count, gastric retention, prolonged QT interval, elevated platelets, decreased hemoglobin, shortness of breath, abdominal pain, joint pain, low back pain, and coagulation abnormalities.

The most common adverse event was fever post-injection, occurring in 36.36% of the patients, followed by excessive salivation (12.12%), cough (12.12%), vomiting (6.06%), and elevated white blood cells (6.06%). A total of 12 patients experienced post-injection fever, including 8 type I SMA patients (66.67%) and 4 type II SMA patients (33.33%). Four patients developed excessive salivation, all of whom were type I SMA patients. Additionally, 4 patients had post-injection cough, with 1 type I SMA patient (25.0%), 2 type II SMA patients (50.0%), and 1 type III SMA patient (25.0%) affected. These results are summarized in Table 5.

**Table 5:** Adverse reactions/events following nusinersen treatment, *n* (%)

Adverse reaction/events	Type I	Type II	Type III	Total	*P*
Adverse reaction, *n* (%)	8(66.67)	8(30.77)	1(25.0)	17(40.48)	0.089
Adverse events, *n* (%)					
Fever	8(66.67)	4(33.33)	0(0)	12(36.36)	0.002*
Excessive Salivation	4(100.0)	0(0)	0(0)	4(12.12)	0.002*
Cough	1(25.0)	2(50.0)	1(25.0)	4(12.12)	0.687
Vomiting	0(0)	2(100.0)	0(0)	2(6.06)	0.05
Elevated WBC Count	2(100.0)	0(0)	0(0)	2(6.06)	0.05
Gastric Retention	1(100.0)	0(0)	0(0)	1(3.03)	0.223
Prolonged QT Interval	1(100.0)	0(0)	0(0)	1(3.03)	0.223
Elevated Platelet Count	1(100.0)	0(0)	0(0)	1(3.03)	0.223
Decreased Hemoglobin	1(100.0)	0(0)	0(0)	1(3.03)	0.223
Shortness of Breath	1(100.0)	0(0)	0(0)	1(3.03)	0.223
Abdominal Pain	0(0)	1(100.0)	0(0)	1(3.03)	0.223
Joint Pain	0(0)	1(100.0)	0(0)	1(3.03)	0.223
Low Back Pain	0(0)	1(100.0)	0(0)	1(3.03)	0.223
Coagulation Abnormalities	0(0)	0(0)	1(100.0)	1(3.03)	0.223
Total	20(60.61)	11(33.33)	2(6.06)	33(100.0)	< 0.001*

Out of the 33 adverse events, the highest incidence rate was observed in type I SMA patients, followed by type II SMA patients (33.33%) and type III SMA patients (6.06%). Statistical analysis revealed significant differences in the incidence of adverse events among the three types of SMA patients (*P* < 0.05).

### Economic evaluation of nusinersen sodium injection for the treatment of SMA

In this study, type III SMA patients have the highest annual cost of nusinersen at 123,198 yuan, followed by type II and type I patients, at 102,035 yuan and 84,687 yuan, respectively. The number of nusinersen injections administered was 9 for type I and type II SMA patients, and 10 for type III SMA patients. The total treatment costs were highest for type III SMA patients, followed by type II and type I SMA patients. The average cost per injection was consistent with the total treatment cost ranking, with no statistically significant differences observed in the economic outcomes across the three SMA types (*P* > 0.05). These results are summarized in Table 6.

**Table 6:** Economic comparison of nusinersen treatment for SMA [M (Q1, Q3)]

Type	Number of injections	Total treatment cost (10000 RMB)	Average cost per injection (10000 RMB)
I	9(7,11)	24.6(18.2,28.3)	2.95(2.13,3.54)
II	9(5,11)	25.8(17.7,28.6)	3.52(2.70,3.57)
III	10(8,12)	31.1(24.3,36.5)	3.55(3.16,3.66)
Total	9(6,11)	25.8(17.8,28.6)	3.51(2.19,3.57)
*P*	0.775	0.541	0.267

## Discussion

SMA is a rare autosomal recessive neurodegenerative disorder that, as it progresses, leads to multisystem impairment, severely impacting the quality of life of affected children and, in severe cases, posing life-threatening risks [21]. Prior to the introduction of nusinersen, SMA patients mainly relied on symptom management or palliative care due to a lack of effective treatment options.

Nusinersen, the first globally and domestically approved disease-modifying therapy for SMA that is effective across all age groups and SMA types, has introduced a significant advancement in SMA treatment. Its mechanism of action involves binding to a specific site downstream of intron 7 of SMN2 mRNA, known as intronic splicing silencer N1, to promote the inclusion of exon 7 in the SMN2 gene. This modification increases the production of functional full-length SMN protein, thereby improving motor function in patients [22]. Due to its inability to cross the blood-brain barrier, Nusinersen must be administered via intrathecal injection to distribute effectively through the cerebrospinal fluid to the target tissues of the central nervous system [23]. Nusinersen has an average terminal elimination half-life in the central nervous system of approximately 135–177 days. The recommended dosage starts with four loading doses (the first three given at 14-day intervals, and the fourth given 30 days after the third), followed by maintenance doses administered every four months.

Nusinersen has now been approved in multiple countries worldwide, where it has demonstrated effectiveness in altering the natural course of SMA in high-evidence-level randomized controlled trials [24, 25]. Furthermore, numerous real-world studies have corroborated these positive results, providing additional support for its beneficial impact in SMA [26, 27]. Regarding safety, several clinical trials have reported adverse reactions associated with nusinersen, including post-lumbar puncture syndrome [24], respiratory adverse events [18, 28], and proteinuria [29]; however, no severe adverse events were noted, and symptoms typically resolved with symptomatic treatment [30].

Currently, there are no reports of pharmacoeconomic studies on nusinersen in China. This study offers a comprehensive analysis of the treatment efficacy, safety, and economic aspects of nusinersen administered intrathecally in 42 SMA children diagnosed and treated at our hospital between January 2022 and October 2024. Our findings aim to provide a basis for the rational clinical use of nusinersen.

The diagnosis of SMA is primarily established through clinical presentation and molecular genetic testing. Over 95% of SMA cases are due to homozygous deletions in exon 7 or exons 7 and 8 of the SMN1 gene [31]. Most patients exhibit homozygous deletions in both exons 7 and 8 of the SMN1 gene, while a smaller proportion display a deletion in only exon 7. In our study, 64.3% of patients had homozygous deletions of exons 7 and 8, while 16.7% had a deletion limited to exon 7. Additionally, three patients presented with a homozygous deletion of exon 7 combined with a heterozygous deletion of exon 8, and two patients had heterozygous deletions of both exons 7 and 8. The three patients with homozygous deletions of exon 7 and heterozygous deletions of exon 8 were all classified as Type II SMA and exhibited relatively mild symptoms, such as developmental delays and inability to stand independently. Following nusinersen treatment, these patients demonstrated significant improvement in motor function. Among the two patients with heterozygous deletions of exons 7 and 8, one was a type I patient who presented with symptoms at 4 months, such as inability to roll over, and showed no significant improvement in motor function post-treatment. The other patient, diagnosed as type III, exhibited symptoms of unsteady standing at 18 months. This patient demonstrated marked improvement in motor function after nusinersen treatment, with clinically meaningful improvements noted in the HFMSE score.

Nusinersen has been shown to improve multiple systemic functions in SMA patients, including respiratory, nutritional, and skeletal functions, and significantly enhances motor function in affected individuals [32]. In clinical practice, motor function improvements following nusinersen treatment are generally assessed using various standardized rating scales.

The changes of motor function in children with SMA can accurately reflect the dynamic changes of the disease. It is suggested that all children should be evaluated for motor function [33]. Adult SMA clinical experts have developed 8 assessment tools for adult SMA outcomes [34], while the assessment of motor function in children needs to selected appropriate scales according to the age and functional status of the child. The CHOP-INTEND scale is recommended for those who cannot sit alone [35], while RULM, HFMSE or MFM-32 are recommended for those who can sit alone. For children aged 2 months to 2 years, we have added the HINE-2 scale to evaluate their developmental milestones [17, 36, 37]. For type I SMA patients, CHOP-INTEND and HINE-2 scores are commonly used to evaluate motor function outcomes [38]. Our study showed that after nusinersen treatment, type I SMA patients experienced the greatest increases in CHOP-INTEND and HINE-2 scores, with mean improvements of 8 points and 3.5 points, respectively. Additionally, these patients had the highest mean increase in HFMSE scores, averaging an improvement of 6.5 points. All three scores demonstrated clinically meaningful improvements.

For type II and III SMA patients, HFMSE and RULM scores are typically used to assess motor function changes. In our study, type II patients exhibited the largest increase in RULM scores, with an average gain of 5.5 points, and an average increase of 4 points in HFMSE scores. Additionally, type II patients showed a mean increase of 8 points in CHOP-INTEND scores, similar to type I patients, and a mean increase of 3 points in HINE-2 scores. All four scores for type II patients indicated clinically meaningful improvements. In contrast, type III patients showed a mean increase of 4 points in HFMSE and 2 points in RULM, both reflecting clinically significant improvements, while CHOP-INTEND and HINE-2 scores increased by an average of 1 point each, which was not clinically significant.

Studies indicate that motor function scales, as primary outcome measures, are more sensitive in younger SMA type I and II patients, with treatment efficacy decreasing with age in older type II and III SMA patients [39]. In our study, post-treatment improvements across all scores in type III SMA patients were lower than those in type I and II patients.

SMA is a progressive neuromuscular disorder, and nusinersen is a gene replacement therapy specifically targeting 5q SMA. Therefore, earlier treatment theoretically offers better efficacy and prognosis. Research has shown that patients treated early, especially those with a disease duration of less than 12 weeks, achieve greater therapeutic benefits [40]. A systematic review evaluating the effectiveness of nusinersen for 5q SMA also demonstrates that earlier treatment, especially pre-symptomatic initiation, yields the best results, often allowing patients to reach normal motor developmental milestones [41]. In this study, type II patients showed the highest proportion of motor function improvement following the first dose of nusinersen, compared to type I and III patients. Two months after the first treatment, however, the proportion of motor function improvement in type III patients was higher than in type I and II patients. Therefore, the sooner SMA is detected and treated with nusinersen, the greater the potential benefits for patients.

Additionally, large-scale newborn SMA screening can facilitate early detection and timely treatment initiation, maximizing therapeutic effects [42]. However, China has yet to implement extensive newborn SMA screening, and nearly all patients are diagnosed only after symptom onset. For symptomatic type I and II patients, improvements in muscle weakness tend to be limited and slow [41].

In summary, the optimal strategy and best cost-effectiveness ratio for treating SMA patients is to implement large-scale newborn SMA screening and initiate nusinersen therapy pre-symptomatically.

The adverse reactions recorded in the nusinersen injection package insert primarily relate to events associated with lumbar puncture, such as headache, back pain, and vomiting. Other adverse reactions include thrombocytopenia, coagulation abnormalities, elevated urinary protein, and hydrocephalus, with relatively low incidence rates. These reactions are likely related to the chemical structure and single nucleotide sequence of the antisense oligonucleotide used in nusinersen, typically resolving on their own after discontinuation of treatment [43]. The FDA recommends regular monitoring for coagulation disorders, thrombocytopenia, and proteinuria in patients receiving nusinersen [44].

Since SMA patients require lifelong lumbar puncture intrathecal administration, lumbar puncture-related adverse events have a high incidence, with post-puncture headache being one of the most common complications. These symptoms are often relieved by lying down, indicating that nusinersen use is relatively safe. Fever and nausea, although not listed in the package insert, are also frequently reported nusinersen-related adverse events [45–47]. Real-world research data from openFDA has shown that headache, post-lumbar puncture syndrome, fever, infectious pneumonia, and vomiting are among the top five reported adverse events [29].

In this study, among the 42 SMA patients analyzed, a total of 33 adverse events were recorded, with post-injection fever having the highest incidence (36.36%)—not post-lumbar puncture syndrome. This could be attributed to the fact that adverse events were more prevalent in type I SMA patients, who had a median age of 33.5 months at treatment initiation. Symptoms of post-lumbar puncture syndrome, such as headache and back pain, largely depend on patient self-report, and younger patients often lack the ability to communicate these symptoms clearly, potentially leading to data bias. Other unlisted adverse reactions observed in this study included cough, elevated white blood cells, gastric retention, prolonged QT interval, elevated platelets, decreased hemoglobin, and abdominal pain. Due to the limited number of patients included, these adverse events warrant clinical attention but require further investigation for confirmation.

In terms of economic research, nusinersen requires six injections in the first year, followed by three injections annually, and treatment must be ongoing, leading to a high overall cost. Current studies on the economic impact of nusinersen for SMA treatment are limited, but reports from the United States, Sweden, and Australia indicate that while nusinersen can improve quality-adjusted life years (QALYs), its high cost limits cost-effectiveness [48–50]. Sensitivity analysis has shown that for nusinersen to meet a $50,000 per QALY threshold, its price would need to be reduced to 19% of the current cost [51].

Currently, no domestic pharmacoeconomic studies on nusinersen are available, though preliminary estimates of its incremental cost-effectiveness ratio (ICER) suggest that even after price reductions, it may still exceed the ICER threshold. However, the reduced cost offers a significant advantage. Data from Zhongkang CHIS further supports this, showing a fivefold increase in nusinersen’s national hospital sales after it entered the healthcare insurance program in the first half of 2022.

The 42 patients included in this study received treatment post-insurance inclusion, indicating a strong willingness to pursue treatment once the cost burden was lowered. Notably, among different SMA types, treatment costs were highest for type III patients, which contrasts with existing literature [51].

The limitations of this study include its short study period, single-center design, and limited sample size. Further data on the drug’s efficacy and safety will require larger sample sizes and extended follow-up periods to provide more reliable data.

## Conclusion

In our study, nusinersen demonstrates improvements in the motor function of patients with various types of SMA, especially in type I patients, with good safety and tolerance profiles. After being covered by healthcare insurance, its economic accessibility has improved considerably, reducing the financial burden on families and enabling long-term treatment for SMA patients. Additionally, large-scale newborn SMA screening as soon as possible can facilitate early detection and timely treatment, and maximize the therapeutic effect.

## Data Availability

No datasets were generated or analysed during the current study.
